# Artificial Intelligence for Non-Invasive Prediction of Molecular Signatures in Spinal Metastases: A Systematic Review

**DOI:** 10.3390/bioengineering12080791

**Published:** 2025-07-23

**Authors:** Vivek Sanker, Sai Sanikommu, Alexander Thaller, Zhikai Li, Philip Heesen, Srinath Hariharan, Emil O. R. Nordin, Maria Jose Cavagnaro, John Ratliff, Atman Desai

**Affiliations:** 1 Department of Neurosurgery, Stanford University, Palo Alto, CA 94305, USA; hsrinath@stanford.edu (S.H.); enordin@stanford.edu (E.O.R.N.); mjcava@stanford.edu (M.J.C.); jratliff@stanford.edu (J.R.); atman@stanford.edu (A.D.); 2 Department of Neurosurgery, Miller School of Medicine, University of Miami, Miami, FL 33136, USA; vms136@miami.edu; 3 Department of Neurosurgery, Medical University of Graz, 8010 Graz, Styria, Austria; alexander.thaller@medunigraz.at; 4 Department of Clinical Neurosciences, Addenbrooke’s Hospital, Cambridge CB2 0QQ, UK; zl498@cam.ac.uk; 5 Faculty of Medicine, University of Zurich, 8032 Zurich, Switzerland; heesenphilip@gmail.com

**Keywords:** artificial intelligence, genetic markers, spinal metastasis, molecular signatures

## Abstract

**Background:** Spinal metastases (SMs) are associated with poor prognosis and significant morbidity. We hypothesize that artificial intelligence (AI) models can enhance the identification and clinical utility of genetic and molecular signatures associated with SMs, improving diagnostic accuracy and enabling personalized treatment strategies. **Methods:** A systematic review of five databases was conducted to identify studies that used AI to predict genetic alterations and SMs outcomes. Accuracy, area under the receiver operating curve (AUC), and sensitivity were used for comparison. Data analysis was performed in R. **Results:** Eleven studies met the inclusion criteria, covering three different primary tumor origins, comprising a total of 2211 patients with an average of 201 ± 90 patients (range: 76–359 patients) per study. EGFR, Ki-67, and HER-2 were studied in ten (90.9%), two (18.1%), and one (9.1%) study, respectively. The weighted average AUC is 0.849 (95% CI: 0.835–0.863) and 0.791 (95% CI: 0.738–0.844) for internal and external validation of the established models, respectively. **Conclusions:** AI, through radiomics and machine learning, shows strong potential in predicting molecular markers in SMs. Our study demonstrates that AI can predict molecular markers in SMs with high accuracy.

## 1. Introduction

The incidence of spinal metastasis is estimated to increase by more than 100,000 cases annually [1,2]. The most common primary sources of spinal metastasis are breast (21%), lung (19%), prostate (7.5%), renal (5%), gastrointestinal (4.5%), and thyroid (2.5%) [3]. Patients may present with pain, spinal instability, pathological fractures, or spinal cord compression with weakness and paralysis [4]. Genetic signatures are the patterns of unique genetic activity or expression seen in certain diseases, including spinal metastasis [5]. These genetic signatures are very helpful in aiding in the diagnosis, prognosis, and treatment of SMs. TP53, PTEN, and RB1, as well as pathways like PI3K, are the most common genetic markers implicated in spinal metastasis, and alterations in these genes and pathways potentially drive the metastatic process [6]. Understanding these genetic and molecular signatures of spinal metastasis is critical for advancing personalized oncological treatment strategies. Such biomarkers can offer insights into tumor behavior and the likelihood of disease progression, treatment resistance, and possible therapeutic targets. Considering the complexity of spinal metastasis, early and precise identification of genetic and molecular signatures can guide tailored treatment strategies, enhance prognostic accuracy, and potentially improve patient outcomes.

Biomarkers such as EGFR, Ki-67, and HER-2 play pivotal roles in spinal metastasis. Epidermal growth factor receptor (EGFR) is a well-studied oncogene with mutations commonly found in non-small cell lung cancer (NSCLC), one of the primary cancers known to cause spinal metastasis [7]. EGFR mutations, especially in exons 19 and 21, are associated with tumor proliferation and responsiveness to tyrosine kinase inhibitors, making EGFR of therapeutic and prognostic relevance in SMs from NSCLC [8]. Ki-67 is linked to cell proliferation and thus serves as a prognostic marker of tumor aggressiveness. Elevated expression, therefore, correlates with rapid growth, poor prognosis, and higher risk of tumor recurrence [9]. Human epidermal growth factor receptor 2 (HER-2) is commonly found in breast cancer, yet its expression has also been identified in spinal metastasis and is associated with increased metastatic potential and targeted treatment responsiveness [10].

Artificial intelligence (AI), including deep learning (DL), machine learning (ML), and various other methods, is being explored to help in the analysis of the vast datasets aiding in research, diagnosis, treatment, and prognosis prediction of various diseases and conditions. Several AI models are being introduced in diagnosing, treating, and prognosing spinal metastasis [11]. AI applications in spinal metastasis utilize the aforementioned techniques as well as radiomics, making use of their distinctive strengths. ML models such as support vector machines (SVMs) and random forests (RFs) are used for classification tasks, e.g., predicting genetic mutations or patient outcomes based on structured clinical or genomic data [12]. SVMs perform well in high-dimensional spaces by identifying optimal hyperplanes for classification [13], while RFs leverage ensemble learning to improve robustness and reduce overfitting [14]. Radiomics refers to the extraction of high-dimensional features from medical imaging, which can be analyzed by ML models for precision diagnosis and treatment [15], or uncover imaging markers associated with molecular alterations. DL models, e.g., artificial neural networks (ANNs) and convolutional neural networks (CNNs), learn hierarchical data representations without the need for much manual input or preprocessing [16]. CNNs are particularly useful in radiomics for automated feature extraction from medical imaging, whereas ANNs can be trained to fully integrate multi-modal inputs [16], improving the prediction of SMs characteristics and outcomes.

In this study, we review and describe the various AI methods used in the literature to identify the genetic signatures associated with SMs.

## 2. Materials and Methods

### 2.1. Ethical Review

Ethical review and approval were waived for this study due to it being a systematic review of previously published data and it did not involve human participants or the collection of new data.

### 2.2. Search Strategy

We searched PubMed, Scopus, Web of Science Advance, Cochrane, and Embase (Ovid) databases to identify relevant studies, using a search query with specific keywords like ‘spine metastases’, ‘artificial intelligence’, ‘machine learning’, ‘deep learning’, ‘neural network’, ‘radiomics’, ‘predictive modeling’, and ‘computer-assisted diagnosis’ (Supplementary Table S1). The population under consideration included adult patients with spinal metastases. The inclusion criterion is original studies in the English language reporting the use of AI/DL models in predicting genetic and molecular signatures in spinal metastases.

The exclusion criterion is as follows: Studies unrelated to spinal metastases and those purely investigating primary spinal tumors. Animal studies, reviews, articles in foreign languages, and non-original research articles were also excluded from our analysis to ensure the inclusion of primary research data relevant to our objective. The electronic search ranged from the period’s earliest available date up to 27 January 2025.

### 2.3. Screening of Studies

**Figure 1:**
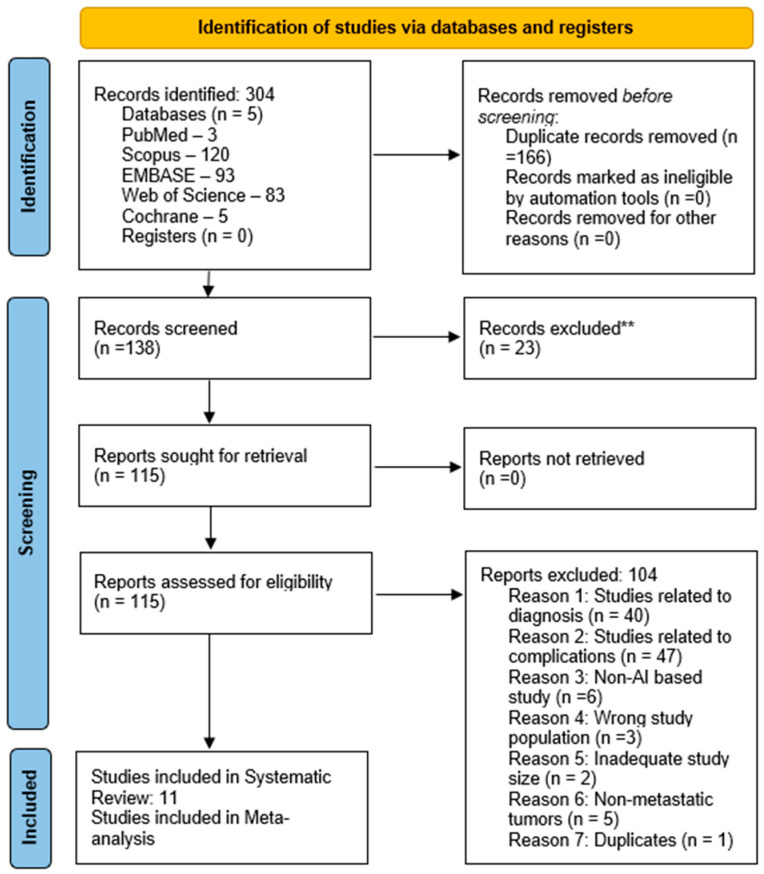
A PRISMA flow diagram is presented to illustrate the screening of studies.

Each study’s title and abstract were screened for relevance before proceeding to full-text screening, which was independently assessed by two reviewers (SS and VS). Any discrepancies were addressed through consultation with a third reviewer (SH). The screening of studies adhered to the PRISMA (preferred reporting items for systematic reviews and meta-analyses) guidelines (Figure 1).

### 2.4. Data Extraction

Three independent authors (SS, VS, and AT) extracted relevant data from the included studies. The data collected included study design, participant demographics, and the number of participants with respective outcomes and complications. Discrepancies in data extraction were resolved through consensus.

### 2.5. Data Analysis

Relevant variables were extracted from each of the included articles, such as the primary tumor type, cohort size, prediction model performance matrices: area under the receiver operating characteristic curve (AUC), and type of validation (internal or external validation). The weighted average of the AUC was calculated. All statistical analyses were conducted using Excel, R Statistical Software, version 4.3.1., and Python, version 3.13.3.

### 2.6. Quality Assessment

The quality assessment was performed using the PROBAST (Prediction model Risk of Bias Assessment Tool) (Supplementary Tables S3 and S4).

PROBAST is designed for assessing the risk of bias and applicability in studies that develop, validate, or update predictive models, which can be analogous to assessing the performance of large language models (LLMs)/natural language processing (NLP) in such settings [17]. It is a structured tool that assesses four domains:

Participants: Evaluating whether the data sources or patient samples used for training and testing are appropriate and representative of the clinical population. Predictors: Ensuring that input data or predictors are well defined and appropriately measured. Outcome: Ensuring that the outcomes (e.g., model predictions, decisions) are clearly defined and relevant to clinical scenarios. Analysis: Evaluating whether the model performance metrics, training/validation processes, and statistical analysis methods are robust and unbiased.

## 3. Results

This review encompasses eleven studies [18,19,20,21,22,23,24,25,26,27,28] published between 2021 and 2024, including three different primary tumor types (non-small cell lung cancer (NSCLC), breast cancer, and lung adenocarcinoma) with a total of 2211 patients (Table 1). Each study included an average of 201 ± 90 patients, ranging from 76 to 359 patients.

Concerning the status of the receptor analyzed (Table 1), one of the eleven studies reported AUC values for the established radiomics models for the training of the model (Table 2), eleven for internal validation (Table 3), and five studies reported AUC values for external validation (Table 4).

The weighted average AUC value among the eleven studies that reported AUC values and corresponding 95% confidence intervals for internal validation of the established models is 0.849 (95% CI: 0.835–0.863). Wherever 95% confidence intervals were not reported, the weighted average of the reported 95% confidence intervals was used.

The weighted average AUC value among the five studies that reported AUC values and corresponding 95% confidence intervals for external validation of the established models is 0.791 (95% CI: 0.738–0.844). Wherever 95% confidence intervals were not reported, the weighted average of the reported 95% confidence intervals was used.

## 4. Discussion

Here, the emerging and significant role of artificial intelligence (AI), particularly radiomics and machine learning (ML), in characterizing genetic and molecular signatures in spinal metastases (SMs) has been highlighted. In the eleven included studies, non-small cell lung cancer (NSCLC) and breast cancer were mainly focused on, with AI models developed that predict biomarkers such as EGFR, HER-2, Ki-67, and T790M. Overall, the models demonstrated promising performance with consistently high AUC values across training and validation cohorts. The ability of AI to analyze complex imaging patterns that may be invisible to clinicians underlines its promise in predicting spinal metastases characteristics. Radiomics—through conversion of medical images into higher-dimensional data—enables quantitative and reproducible analyses that can predict tumor biology non-invasively when combined with ML algorithms. This is invaluable in spinal metastases, where biopsy is often technically challenging and risky due to the anatomical proximity of critical neurovascular structures.

EGFR mutation status emerged as the most commonly predicted biomarker across the included studies, especially in spinal metastases from primary NSCLC. For instance, Hu et al. [18] achieved an AUC of 0.929 in training and 0.865 in external validation using CT and MRI-derived radiomics from both primary and metastatic lesions. Additionally, Cheng et al. [19] demonstrated that combining radiomics features from both primary lung tumors and spinal metastases enhanced prediction of EGFR mutations and response to EGFR-tyrosine kinase inhibitor therapy, with external validation AUCs > 0.80. These findings underline the ability of AI to effectively detect the distinct and clinically relevant molecular profiles that metastatic lesions carry.

Other biomarkers aside from EGFR, such as Ki-67 and HER-2, were also predicted successfully. Zhang et al. [23] used MRI-based features to forecast Ki-67 and HER-2 status in breast cancer spinal metastases, achieving AUCs near or above 0.80 in both training and external validation data sets. The high-performance metrics underscore the robustness and reproducibility of these AI tools, justifying their clinical utility for treatment stratification and prognostication. Importantly, external validation was present in most studies and enhances finding generalizability. Nonetheless, there were performance drop-offs between internal and external cohorts, which is typical in AI research and reflects variability in imaging protocols, scanner types, and patient populations across centers. Such variability proves that multicentric datasets and model standardization are needed to bridge the translational gap from research to real-world clinical application.

The inclusion of deep learning models, such as CM-EfNet by Jiang et al. [21], which integrates advanced modules like the convolutional block attention model (CBAM) and multi-resolution feature fusion, represents a crucial next step in enhancing prediction accuracy and computational efficiency. These models performed superiorly over traditional radiomics with external AUCs reaching up to 0.764 (95%CI: 0.615–0.914), highlighting the evolving sophistication of AI techniques.

Clinically, non-invasive prediction of actionable mutations in SMs may potentially facilitate personalized therapy without necessitating repeated tissue biopsies. This could be particularly relevant in tracking resistance mutations such as T790M in lung adenocarcinoma, as demonstrated in studies by Fan et al. [24,25] and Cao et al. [22], where AI-derived radiomics nomograms showed AUCs above 0.80.

The integration of AI models into routine clinical practice and patient care for SMs remains promising yet faces significant hurdles due to several healthcare and technological constraints. Challenges include a lack of standardized, high-quality datasets, limited interoperability with clinical systems, and concerns over model transparency, interpretability, and generalizability [29,30]. In addition, regulatory approval, general data protection regulations, and clinicians’ acceptance and knowledge of AI models pose as roadblocks. Despite this, advances in explainable AI and collaborative data sharing efforts are gradually improving the feasibility of widespread clinical adoption and use [31].

Despite these encouraging results, certain limitations must be acknowledged. First, most studies had relatively small sample sizes (average cohort size: ~200 patients) and resultant overfitting can inflate performance metrics. Second, most data were from Chinese populations, questioning the models’ applicability in other ethnic and geographic groups. Third, only a few studies incorporated clinical variables (e.g., smoking history or performance status), which could further enrich model performance and interpretability. Fourth, while PROBAST assessment indicated generally low to moderate risk of bias across domains, the heterogeneity in model architectures, radiomics feature sets, and validation strategies precluded direct comparison of model efficacy. Shared open-access datasets and consensus on performance reporting standards, which have been promoted in other areas of AI in oncology, would likely be beneficial.

## 5. Conclusions

AI models have shown good potential in predicting key molecular markers in spinal metastases with high accuracy, permitting a preferable non-invasive alternative to biopsies, for both identifying actionable mutations like EGFR and T790M and also in stratifying patients on the basis of biomarkers such as Ki-67 and HER-2 to provide more customized and timely clinical decision-making. However, to further integrate these key tools into daily practice, further large, multi-center datasets reflecting global diversity and differential epidemiology and demographics must be curated, along with standardized radiomics workflows, and multimodal AI models, handling clinical and genomic features, to enhance utility.

## Figures and Tables

**Table 1:** An overview of the studies analyzed is presented.

Study	Cohort Size	Mean Age ± SD	Receptor Analyzed	Primary Tumor Type
Hu et al. (2024) [18]	359	Training cohort = 58.21 ± 9.28 Validation cohort = 59.87 ± 7.23	EGFR	NSCLC
Cheng et al. (2023) [19]	203	Training cohort (EGFR mutant) = 58.12 ± 9.62 (EGFR wild type) = 58.82 ± 10.14 Validation cohort (EGFR mutant) = 58.41 ± 9.04 (EGFR wild type) = 60.35 ± 9.61	EGFR	NSCLC
Niu et al. (2023) [20]	268	NSCLC = 57.88 ± 10.81 Breast cancer = 53.71 ± 9.77	EGFR, Ki-67	NSCLC Breast cancer
Jiang et al. (2023) [21]	265	Training cohort (EGFR-21) = 61.91 ± 10.75 (EGFR 19) = 57.43 ± 9.34 (EGFR wild type) = 59.18 ± 9.99 Validation cohort (EGFR-21) = 61.54 ± 10.85 (EGFR 19) = 58.71 ± 9.72 (EGFR wild type) = 60.13 ± 7.38	EGFR	NSCLC
Cao et al. (2023) [22]	299	Training cohort (EGFR mutant) = 60.19 ± 9.99 (EGFR wildtype) = 59.28 ± 10.35 Validation cohort (internal) (EGFR mutant) = 61.15 ± 11.77 (EGFR wildtype) = 59.60 ± 10.42 (external) (EGFR mutant) = 60.17 ± 7.63 (EGFR wildtype) = 60.06 ± 6.78	EGFR	Lung adenocarcinoma
Zhang et al. (2024) [23]	110	Training cohort (High Ki-67) = 54.48 ± 9.76 (Low Ki-67) = 54.31 ± 9.06 (HER-2 positive) = 49.73 ± 11.82 (HER-2 negative) = 53.90 ± 8.70 Validation cohort (High Ki-67) = 52.50 ± 9.22 (Low Ki-67) = 49.06 ± 9.51 (HER-2 positive) = 49.01 ± 9.23 (HER-2 negative) = 56.57 ± 10.01	HER-2, Ki-67	Breast cancer
Fan et al. (2022) [24]	183	Training cohort (EGFR mutant) = 58.71 ± 9.34 (EGFR wildtype) = 57.14 ± 11.28 Validation cohort (internal) (EGFR mutant) = 58.14 ± 12.11 (EGFR wildtype) = 59.21 ± 8.95 (external) (EGFR mutant) = 59.06 ± 7.78 (EGFR wildtype) = 60.14 ± 5.61	EGFR	Lung adenocarcinoma
Fan et al. (2022) [25]	192	Training cohort (EGFR mutant) = 58.69 ± 10.31 (EGFR wildtype) = 58.37 ± 9.59 Validation cohort (internal) (EGFR mutant) = 61.5 ± 7.39 (EGFR wildtype) = 56.88 ± 10.10 (external) (EGFR mutant) = 63.06 ± 9.11 (EGFR wildtype) = 60.86 ± 6.64	EGFR	Lung adenocarcinoma
Cao et al. (2022) [26]	76	Training cohort (EGFR-21) = 61.12 ± 11.45 (EGFR 19) = 59.44 ± 8.65 Validation cohort (EGFR-21) = 62.69 ± 11.44 (EGFR 19) = 53.54 ± 10.70	EGFR	Lung adenocarcinoma
Fan et al. (2021) [27]	94	Training cohort (EGFR mutant) = 58.52 ± 9.84 (EGFR wild type) = 61.70 ± 10.75 Validation cohort (EGFR mutant) = 57.26 ± 9.43 (EGFR wild type) = 57.08 ± 10.66	EGFR	Lung adenocarcinoma
Ren et al. (2021) [28]	162	Training cohort (EGFR mutant) = 60.60 ± 10 (EGFR wild type) = 59.10 ± 11.20 Validation cohort (EGFR mutant) = 61.10 ± 9.29 (EGFR wild type) = 60.00 ± 6.64	EGFR	Lung adenocarcinoma

**Table 2:** The AUC and corresponding 95% confidence interval of studies reporting AUC values for the established radiomics models for the training of the model are presented. 1 = epidermal growth factor receptor.

Study	Output/Prediction	Best Performing Model	AUC	95% CI
Hu et al. (2024) [18]	EGFR ^1^ mutation status	Radiomics (RS-SM-Com)	0.929	0.886–0.973

**Table 3:** The AUC and corresponding 95% confidence interval of studies reporting AUC values for the established radiomics models for internal validation of the model is presented. 1 = epidermal growth factor receptor, 2 = k-fold cross validation, 3 = split sample internal validation, 4 = EGFR ^1^-tyrosine kinase inhibitor, 5 = non-small cell lung cancer, 6 = breast cancer, 7 = convolutional block attention module, 8 = multi-resolution feature fusion mechanism, 9 = human epidermal growth factor receptor 2.

Study	Output/Prediction	Best Performing Model	AUC		95% CI	
Hu et al. (2024) [18]	EGFR ^1^ mutation status	Radiomics (RS-SM-Com)	0.896		0.781–1.000	
Cao et al. (2023) [22]	EGFR ^1^ mutation status	Radiomics (RS-Com-EGFR)	0.806 ^2^	0.745 ^3^	-	-
	EGFR ^1^ Exon 19 mutation	Radiomics (RS-Com-Exon19)	0.872 ^2^	0.760 ^3^	-	-
	EGFR ^1^ Exon 21 mutation	Radiomics (RS-Com-Exon21)	0.913 ^2^	0.799 ^2^	-	-
Cheng et al. (2023) [19]	EGFR ^1^ mutation status	Radiomics (RS-Com-EGFR)	0.927 ^2^	0.812 ^3^	0.884–0.969 ^2^	0.709–0.916 ^3^
	Response to EGFR-TKI ^4^	Radiomics (RS-Com-TKI)	0.880 ^2^	0.798 ^3^	0.802–0.958 ^2^	0.649–0.946 ^3^
Jiang et al. (2023) [21]	EGFR ^1^ mutation status	CM-EfNet (CBAM ^7^ and MFM ^8^ and EfficientNet v2)	0.866 ^2^	0.851 ^3^	0.800–0.916 ^2^	0.750–0.923 ^3^
	EGFR ^1^ mutations in Exons 19 and 21	CM-EfNet (CBAM ^7^ and MFM ^8^ and EfficientNet v2)	0.760 ^2^	0.711 ^3^	0.656–0.846 ^2^	0.552–0.839 ^3^
Niu et al. (2023) [20]	Differentiating NSCLC ^5^ and BC ^6^ spinal metastasis	Radiomics—logistic regression (Ori-RS)	0.890 ^2^	0.881 ^3^	0.843–0.938 ^2^	0.810–0.953 ^3^
	EGFR ^1^ mutation status	Radiomics—logistic regression (EGFR-RS)	0.793 ^2^	0.744 ^3^	0.703–0.833 ^2^	0.601–0.887 ^3^
	Ki-67 expression level	Radiomics—logistic regression (Ki-67-RS)	0.798 ^2^	0.738 ^3^	0.693–0.902 ^2^	0.554–0.921 ^3^
Zhang et al. (2024) [23]	Ki-67 level	Radiomics (RS-Ki-67)	0.812 ^2^	0.799 ^3^	0.710–0.914 ^2^	0.652–0.947 ^3^
	HER-2 ^9^ mutation status	Radiomics (RS-HER-2)	0.796 ^2^	0.705 ^3^	0.686–0.906 ^2^	0.506–0.904 ^3^
Cao et al. (2022) [26]	Differentiating Exon 19 and Exon 21 in EGFR ^1^ mutation	Nomogram	0.901 ^2^	0.882 ^3^	0.783–0.967 ^2^	0.695–0.974 ^3^
Fan et al. (2022) [24]	EGFR ^1^ mutation status	Radiomics (RS-EGFR)	0.851 ^2^	0.780 ^3^	0.774–0.921 ^2^	0.645–0.916 ^3^
	EGFR ^1^ Exon 19 deletion	Radiomics (RS-19)	0.816 ^2^	0.789 ^3^	0.716–0.917 ^2^	0.636–0.942 ^3^
	EGFR ^1^ Exon 21 mutation	Radiomics (RS-21)	0.814 ^2^	0.770 ^3^	0.714–0.914 ^2^	0.609–0.931 ^3^
Fan et al. (2022) [25]	EGFR ^1^ mutation status	Clinical-radiomics nomogram model (nomogram-EGFR)	0.849 ^2^	0.828 ^3^	0.776–0.922 ^2^	0.708–0.949 ^3^
	T790M mutation status	Clinical-radiomics nomogram model (nomogram-T790M)	0.842 ^2^	0.823 ^3^	0.717–0.927 ^2^	0.633–0.940 ^3^
Fan et al. (2021) [27]	EGFR ^1^ mutation status	Radiomics (multi-regional radiomics signature)	0.879 ^2^	0.777 ^3^	0.766–0.947 ^2^	0.612–0.967 ^3^
Ren et al. (2021) [28]	EGFR ^1^ mutation status	Combined rad score	0.886 ^2^	0.803 ^3^	0.826–0.947 ^2^	0.682–0.924 ^3^

**Table 4:** The AUC and corresponding 95% confidence interval of studies reporting AUC values for the established radiomics models for external validation of the model are presented. 1 = epidermal growth factor receptor, 7 = convolutional block attention module, 8 = multi-resolution feature fusion mechanism.

Study	Output/Prediction	Best Performing Model	AUC	95% CI
Hu et al. (2024) [18]	EGFR mutation status	Radiomics (RS-SM-Com)	0.865	0.731–0.998
Cao et al. (2023) [22]	EGFR mutation status	Radiomics (RS-Com-EGFR)	0.738	-
	EGFR Exon 19 mutation	Radiomics (RS-Com-Exon19)	0.825	-
	EGFR Exon 21 mutation	Radiomics (RS-Com-Exon21)	0.811	-
Jiang et al. (2023) [21]	EGFR mutation status	CM-EfNet (CBAM ^7^ and MFM ^8^ and EfficientNet v2)	0.764	0.615–0.914
	EGFR mutations in Exons 19 and 21	CM-EfNet (CBAM ^7^ and MFM ^8^ and EfficientNet v2)	0.687	0.476–0.897
Fan et al. (2022) [24]	EGFR mutation status	Radiomics (RS-EGFR)	0.807	0.595–0.938
	EGFR Exon 19 deletion	Radiomics (RS-19)	0.742	0.478–0.919
	EGFR Exon 21 mutation	Radiomics (RS-21)	0.792	0.530–0.946
Fan et al. (2022) [25]	EGFR ^1^ mutation status	Clinical-radiomics nomogram model (nomogram-EGFR)	0.778	0.610–0.946
	T790M mutation status	Clinical-radiomics nomogram model (nomogram-T790M)	0.800	0.548–0.948

## Data Availability

All datasets used for this study are presented in the manuscript and its Supplementary Materials.

## Supplementary Materials

The following supporting information can be downloaded at https://www.mdpi.com/article/10.3390/bioengineering12080791/s1, Supplementary Table S1. Search queries across five databases (PubMed, Scopus, Web of Science Advance, Cochrane and Embase (Ovid)) are shown; Supplementary Table S2. A summary of the studies focusing on genetic and molecular signatures in spinal metastases is presented; Supplementary Table S3. PROBAST Step 1—specifying the systematic review question; Supplementary Table S4. PROBAST Step 2—classifying the type of prediction model evaluation; Supplementary Figure S1. Risk of bias; Supplementary Figure S2. Applicability.
