# Supplementary material for: Artificial Intelligence for Non-Invasive Prediction of Molecular Signatures in Spinal Metastases: A Systematic Review
Source: Bioengineering (Basel). 2025 Jul 23;12(8):791. doi: 10.3390/bioengineering12080791 (PMC12383027; doi:10.3390/bioengineering12080791)

bioengineering

MDPI

Supplementary Files

Supplementary Table S1. Search queries across five databases (PubMed, Scopus, Web of Science Advance, Cochrane and Embase (Ovid)) are shown.

Database	No	Search Query	Results
EMBASE			
	#1	#2 AND **'article'**/it<br>**#1** (**'artificial intelligence'**/exp OR **'artificial intelligence'** OR **'machine learning'**/exp OR **'machine learning'** OR **'deep learning'**/exp OR **'deep learning'** OR **'neural network'**/exp OR **'neural network'** OR **'artificial intelligence'**:ti,ab OR **'machine learning'**:ti,ab OR **'deep learning'**:ti,ab OR **'neural network'**:ti,ab OR **'radiomics'**:ti,ab OR **'predictive modeling'**:ti,ab OR **'computer assisted diagnosis'**:ti,ab) AND (**'spinal metastasis'**/exp OR **'spinal metastasis'** OR **'spine metastasis'**:ti,ab OR **'spinal metastasis'**:ti,ab OR **'metastatic spinal disease'**:ti,ab OR **'vertebral metastasis'**:ti,ab)	93
PubMed			
	#1	(("artificial intelligence"[MeSH Terms] OR "machine learning"[MeSH Terms] OR "deep learning"[MeSH Terms] OR "artificial intelligence"[Title/Abstract] OR "machine learning"[Title/Abstract] OR "deep learning"[Title/Abstract] OR "neural networks"[Title/Abstract] OR "radiomics"[Title/Abstract] OR "computer-assisted diagnosis"[Title/Abstract] OR "predictive modeling"[Title/Abstract]) AND ("spinal metastasis"[Title/Abstract] OR "metastatic spinal disease"[Title/Abstract] OR "spine metastasis"[Title/Abstract] OR "vertebral metastasis"[Title/Abstract] OR "metastatic spine disease"[Title/Abstract])) AND ((excludepreprints[Filter] OR medline[Filter]) AND (casereports[Filter] OR classicalarticle[Filter] OR clinicalstudy[Filter] OR clinicaltrial[Filter] OR multicenterstudy[Filter] OR observationalstudy[Filter] OR randomizedcontrolledtrial[Filter] OR technicalreport[Filter]) AND (humans[Filter]) AND (female[Filter] OR male[Filter]) AND (english[Filter]))	3
Scopus			
	#1	TITLE-ABS-KEY ("artificial intelligence" OR "machine learning" OR "deep learning" OR "neural network" OR "radiomics" OR "predictive modeling" OR "computer-assisted diagnosis" OR "AI-based diagnosis") AND TITLE-ABS-KEY ("spinal metastasis" OR "spinal metastases" OR "metastatic spinal disease" OR "spine metastasis" OR "vertebral metastasis") AND (LIMIT-TO (LANGUAGE , "English")) AND (LIMIT-TO (DOCTYPE , "ar"))	120
Web of Science			

	#1	TS=("artificial intelligence" OR "machine learning" OR "deep learning" OR "neural network" OR "radiomics" OR "predictive modeling" OR "computer-assisted diagnosis" OR "AI-based diagnosis") AND TS=("spinal metastasis" OR "spinal metastases" OR "metastatic spinal disease" OR "spine metastasis" OR "vertebral metastasis") and Article (Document Types)	83
Cochrane			
	#1	("artificial intelligence" OR "machine learning" OR "deep learning" OR "neural networks" OR "radiomics" OR "predictive modeling" OR "computer-assisted diagnosis" OR "AI-based diagnosis") AND ("spinal metastasis" OR "spinal metastases" OR "metastatic spinal disease" OR "spine metastasis" OR "vertebral metastasis")	5

Supplementary Table S2 – A summary of the studies focusing on genetic and molecular signatures in spinal metastases is presented.

Sl No	Title	Year	Authors	Primary Tumor Type	Cohort Size	Receptor/ Molecules Involved	Model	CT-based Features	MRI-based Features	Output/ Prediction	Accuracy	Sensitivity	Specificity	AUC	Summary
1	Improved Prediction of Epidermal Growth Factor Receptor Status by Combined Radiomics of Primary Non-small-Cell Lung Cancer and Distant Metastasis	2024	Hu, Y. et al. [18]	Non-Small Cell Lung Cancer (NSCLC)	290 (Primary), 69 (External)	Epidermal Growth Factor Receptor (EGFR) mutation	Machine Learning (Radiomics)	6 CT-based features (3 intra tumoral, 3 peri tumoral)	MRI-based features from brain and spinal metastases	EGFR mutation status	Training:<br><br>RS-SM Com 0.919<br><br>Internal Validation Cohort:<br><br>RS-SM Com 0.857<br><br>External Validation Cohort:<br><br>RS-SM Com 0.786	Training:<br><br>RS-SM Com 0.828<br><br>Internal Validation Cohort:<br><br>RS-SM Com 0.812<br><br>External Validation Cohort:<br><br>RS-SM Com 0.737	Training:<br><br>RS-SM Com 0.875<br><br>Internal Validation Cohort:<br><br>RS-SM Com 0.917<br><br>External Validation Cohort:<br><br>RS-SM Com 0.929	Training Cohort:<br><br>RS-SM Com 0.929 (0.886–0.973)"<br><br>Internal Validation Cohort:<br><br>RS-SM Com 0.896 (0.781–1.000)<br><br>External Validation Cohort:<br><br>RS-SM Com 0.865 (0.731–0.998)	Radiomic signatures (RS-BM-Com and RS-SM-Com) integrating primary and metastatic tumor features enhanced EGFR mutation prediction.
2	Combined radiomics of primary tumour and bone metastasis improve the prediction of EGFR mutation status and response to EGFR-TKI therapy for NSCLC	2023	Cheng, Y. et al. [19]	Non-Small-Cell Lung Cancer (NSCLC)	203	EGFR Mutation	Machine Learning (Radiomics)	Extracted radiomics features from CT images of primary tumor	Extracted radiomics features from MRI images of spinal metastases	EGFR mutation status and therapeutic response to EGFR-TKI	EGFR mutation status.<br>Training group RS-Com-EGFR 0.867<br>Validation group RS-Com-EGFR 0.750<br>EGFR-TKI.<br>Training group RS-Com-TKI 0.784<br>Validation group	EGFR mutation status.<br>Training group RS-Com-EGFR 0.945<br>Validation group RS-Com-EGFR 0.622<br>EGFR-TKI.<br>Training group RS-Com-TKI 0.906<br>Validation group RS-Com-TKI 0.625	EGFR mutation status.<br>Training group RS-Com-EGFR 0.774<br>Validation group RS-Com-EGFR 0.903<br>EGFR-TKI.<br>Training group RS-Com-TKI 0.756<br>Validation group RS-Com-TKI 0.905	(EGFR mutation status.<br>Training group RS-Com-EGFR 0.927 (0.884–0.969)<br>Internal Validation group RS-Com-EGFR 0.812 (0.709–0.916)<br>EGFR-TKI.<br>Training group RS-Com-TKI 0.880 (0.802–0.958)	Integrated primary tumor and metastases for more accurate prediction of EGFR mutation status and therapeutic response to EGFR-TKI in metastatic NSCLC patients.

											RS-Com-TKI 0.784			Internal Validation group RS-Com-TKI 0.798 (0.649–0.946)	
3	Radiomics of Spinal Metastases Originating from Primary Non-small Cell Lung Cancer or Breast Cancer and Ability to Predict Epidermal Growth Factor Receptor Mutation/Ki-67 Levels	2023	Niu, S. et al. [20]	NSCLC and Breast Cancer	268 patients (148 NSCLC, 120 BC)	EGFR Mutation, Ki-67 Expression	Machine Learning (LASSO Regression)	No	Contrast-enhanced T1-weighted MRI	Origin of metastasis, EGFR mutation status, Ki-67 level	NA	NA	NA	Training: Ori-RS :0.890 (0.843-0.938), EGFR-RS :0.793 (0.703-0.833), Ki-67-RS: 0.798 (0.693-0.902) Validation: Ori-RS :0.881 (0.810-0.953), EGFR-RS :0.744 (0.601-0.887), Ki-67-RS: 0.738 (0.554-0.921)	This study developed MRI-based radiomics models to differentiate spinal metastases from NSCLC and BC and predict EGFR mutation and Ki-67 expression. Three radiomics signatures (Ori-RS, EGFR-RS, and Ki-67-RS) were created, with Ori-RS achieving the highest performance in both training and validation cohorts. These models demonstrate potential to guide personalized treatment strategies.
4	Deep learning for preoperative prediction of the EGFR mutation and subtypes based on the MRI image of spinal metastasis from primary NSCLC	2023	Jiang, T. et al. [21]	NSCLC (EGFR Mutation Prediction from Bone Metastases)	Not specified Primary cohort 223, Validation cohort 42	Not specified EGFR Mutation	Deep Learning (CM-EfNet with CBAM + MFM + Efficient Net v2)	MRI-based deep learning features	EGFR Mutation (Positive/Negative) & Mutation Subtype (Exon 19 vs. Exon 21)	NA	Radiomics Training 0.698 Internal validation 0.620; External validation 0.645 EfficientNetv2 Training 0.797; Internal validation 0.773; External validation 0.714	Radiomics Training 0.693; Internal validation 0.535; External validation 0.706 EfficientNetv2 0.729; Internal validation 0.744; External validation 0.500 CM-EfNet Training 0.824 Internal validation 0.767	Radiomics Training 0.680 Internal validation 0.627 External validation 0.677 EfficientNetv2 Training 0.889 Internal validation 0.812 External validation 0.846 CM-EfNet Training 0.825 Internal validation 0.906	EGFR Mutation Status: Radiomics Training (k-fold cross validation) 0.725 (0.646–0.806) Internal validation 0.579 (0.448–0.710) External validation 0.698 (0.547–0.850) EfficientNetv2 Training 0.844 (0.775–0.898) Internal	The study introduces CM-EfNet, integrating convolutional block attention module (CBAM) and multi-resolution feature fusion (MFM) with Efficient Net v2, for end-to-end EGFR mutation detection in metastatic

											CM-EfNet Training 0.824; Internal validation 0.826; External validation 0.786	External validation 0.812	External validation 0.769	validation 0.807 (0.699–0.889) External validation 0.680 (0.509–0.851) CM-EfNet Training 0.866 (0.800–0.916) Internal validation 0.851 (0.750–0.923) External validation 0.764 (0.615–0.914)<br><br>EGFR mutations in Exon 19 and 21: CM-EfNet Training 0.760 (0.656-0.846) Internal validation 0.711 (0.552-839) External validation 0.687 (0.476-0.897)	NSCLC. The proposed deep learning model outperforms traditional radiomics-based approaches, offering a non-invasive imaging tool for preoperative mutation prediction.
5	Comprehensive analysis of prediction of the EGFR mutation and subtypes based on the spinal metastasis from primary lung adenocarcinoma	2023	Cao, R. et al. [22]	Lung Adenocarcinoma with Spinal Metastasis	257 (primary) + 42 (external)	EGFR mutation (Exon 19, Exon 21) Exon 18/20	Machine Learning (5-fold cross-validation)	No	Yes (T1W, T2FS MRI)	EGFR mutation and subtype prediction (Exon 19, Exon 21)	Training: RS-EGFR-T1W: 0.740 RS-EGFR-T2FS: 0.716 RS-Com-EGFR: 0.732 RS-Exon 19-T1W: 0.737 RS-Exon 19-T2FS: 0.702 RS-Com-Exon 19: 0.762 RS-Exon 21-T1W: 0.781 RS-Exon 21-T2FS: 0.769 RS-Com-Exon 21: 0.780<br><br>Internal Validation: RS-EGFR-T1W: 0.738	Training: RS-EGFR-T1W: 0.714 RS-EGFR-T2FS: 0.754 RS-Com-EGFR: 0.670 RS-Exon 19-T1W: 0.784 RS-Exon 19-T2FS: 0.863 RS-Com-Exon 19: 0.922 RS-Exon 21-T1W: 0.812 RS-Exon 21-T2FS: 0.812 RS-Com-Exon 21: 0.875 Internal Validation: RS-EGFR-T1W: 0.652	Training: RS-EGFR-T1W: 0.725 RS-EGFR-T2FS: 0.688 RS-Com-EGFR: 0.863 RS-Exon 19-T1W: 0.775 RS-Exon 19-T2FS: 0.700 RS-Com-Exon 19: 0.700 RS-Exon 21-T1W: 0.767 RS-Exon 21-T2FS: 0.721 RS-Com-Exon 21: 0.837 Internal Validation: RS-EGFR-T1W: 0.850	Training (k-fold cross validation): RS-EGFR-T1W: 0.756 RS-EGFR-T2FS: 0.753 RS-Com-EGFR: 0.806 RS-Exon 19-T1W: 0.840 RS-Exon 19-T2FS: 0.828 RS-Com-Exon 19: 0.872 RS-Exon 21-T1W: 0.854 RS-Exon 21-T2FS: 0.835 RS-Com-Exon 21: 0.913 Internal Validation:	Multi-parametric MRI-based radiomics combined with clinical factors successfully predicted EGFR mutations and subtypes in lung adenocarcinoma spinal metastases. Nomogram models (integrating radiomics and clinical features) outperformed radiomics-only and clinical models.

											RS-EGFR-T2FS: 0.650<br>RS-Com-EGFR: 0.704<br>RS-Exon 19-T1W: 0.720<br>RS-Exon 19-T2FS: 0.715<br>RS-Com-Exon 19: 0.758<br>RS-Exon 21-T1W: 0.721<br>RS-Exon 21-T2FS: 0.690<br>RS-Com-Exon 21: 0.771<br>External Validation:<br>RS-EGFR-T1W: 0.698<br>RS-EGFR-T2FS: 0.672<br>RS-Com-EGFR: 0.709<br>RS-Exon 19-T1W: 0.711<br>RS-Exon 19-T2FS: 0.709<br>RS-Com-Exon 19: 0.736<br>RS-Exon 21-T1W: 0.719<br>RS-Exon 21-T2FS: 0.713<br>RS-Com-Exon 21: 0.723	RS-EGFR-T2FS: 0.697<br>RS-Com-EGFR: 0.696<br>RS-Exon 19-T1W: 0.692<br>RS-Exon 19-T2FS: 0.731<br>RS-Com-Exon 19: 0.962<br>RS-Exon 21-T1W: 0.792<br>RS-Exon 21-T2FS: 0.746<br>RS-Com-Exon 21: 0.750<br>External Validation:<br>RS-EGFR-T1W: 0.679<br>RS-EGFR-T2FS: 0.724<br>RS-Com-EGFR: 0.687<br>RS-Exon 19-T1W: 0.742<br>RS-Exon 19-T2FS: 0.857<br>RS-Com-Exon 19: 0.869<br>RS-Exon 21-T1W: 0.807<br>RS-Exon 21-T2FS: 0.736<br>RS-Com-Exon 21: 0.786	RS-EGFR-T2FS: 0.696<br>RS-Com-EGFR: 0.725<br>RS-Exon 19-T1W: 0.750<br>RS-Exon 19-T2FS: 0.750<br>RS-Com-Exon 19: 0.550<br>RS-Exon 21-T1W: 0.727<br>RS-Exon 21-T2FS: 0.708<br>RS-Com-Exon 21: 0.864<br>External Validation:<br>RS-EGFR-T1W: 0.771<br>RS-EGFR-T2FS: 0.697<br>RS-Com-EGFR: 0.755<br>RS-Exon 19-T1W: 0.731<br>RS-Exon 19-T2FS: 0.728<br>RS-Com-Exon 19: 0.739<br>RS-Exon 21-T1W: 0.732<br>RS-Exon 21-T2FS: 0.729<br>RS-Com-Exon 21: 0.714	RS-EGFR-T1W: 0.742<br>RS-EGFR-T2FS: 0.696<br>RS-Com-EGFR: 0.745<br>RS-Exon 19-T1W: 0.758<br>RS-Exon 19-T2FS: 0.754<br>RS-Com-Exon 19: 0.760<br>RS-Exon 21-T1W: 0.758<br>RS-Exon 21-T2FS: 0.722<br>RS-Com-Exon 21: 0.799<br>External Validation:<br>RS-EGFR-T1W: 0.729<br>RS-EGFR-T2FS: 0.697<br>RS-Com-EGFR: 0.738<br>RS-Exon 19-T1W: 0.819<br>RS-Exon 19-T2FS: 0.801<br>RS-Com-Exon 19: 0.825<br>RS-Exon 21-T1W: 0.804<br>RS-Exon 21-T2FS: 0.713<br>RS-Com-Exon 21: 0.811	
6	Radiomics signatures for predicting the Ki-67 level and HER-2 status based on bone metastasis from primary breast cancer	2023	Zhang, H. et al. [23]	Breast Cancer with Spinal Metastasis	110	Ki-67, HER-2	Machine Learning (LASSO-based feature selection)	No	Yes (T1-Weighted Contrast-Enhanced MRI)	Ki-67 proliferation index and HER-2 status prediction	N/A	Ki-67 model sensitivity: Training 0.667, Validation 0.722<br>HER-2 model sensitivity: Training 0.720, Validation 0.733	Ki-67 model specificity: Training 0.846, Validation 0.833<br>HER-2 model specificity: Training 0.776, Validation 0.762	Ki-67 model AUC: Training (k-fold cross validation) 0.812 (95% CI: 0.710–0.914), Internal Validation 0.799 (95% CI: 0.652–0.947)	MRI-based radiomics features were used to develop models predicting Ki-67 levels and HER-2 status in spinal metastases from breast cancer. Good predictive

														HER-2 model AUC: Training (k-fold cross validation) 0.796 (95% CI: 0.686–0.906), Internal Validation 0.705 (95% CI: 0.506–0.904)	performance was achieved, with high AUC values in both training and validation cohorts.
7	Development and validation of MRI-based radiomics signatures as new markers for preoperative assessment of EGFR mutation and subtypes from bone metastases	2022	Fan, Y. et al. [24]	Lung Adenocarcinoma with Spinal Metastasis	183 (159 primary + 24 external)	EGFR mutation (Exon 19, Exon 21, other mutations	Machine Learning (LASSO-based feature selection)	No	Yes (CE T1-Weighted MRI)	EGFR mutation and subtype prediction (Exon 19 deletion, Exon 21 L858R mutation)	Primary Training: 0.800 (RS-EGFR), 0.739 (RS-19), 0.739 (RS-21) Internal Validation: 0.741 (RS-EGFR), 0.600 (RS-19), 0.657 (RS-21) External Validation: 0.625 (RS-EGFR), 0.623 (RS-19), 0.529 (RS-21)	Primary Training: 0.722 (RS-EGFR), 0.964 (RS-19), 0.655 (RS-21) Internal Validation: 0.771 (RS-EGFR), 0.786 (RS-19), 0.800 (RS-21) External Validation: 0.765 (RS-EGFR), 0.833 (RS-19), 0.750 (RS-21)	Primary Training: 0.870 (RS-EGFR), 0.561 (RS-19), 0.875 (RS-21) Internal Validation: 0.737 (RS-EGFR), 0.714 (RS-19), 0.700 (RS-21) External Validation: 0.857 (RS-EGFR), 0.636 (RS-19), 0.889 (RS-21)	Primary Training (k-fold cross validation): 0.851 (0.774-0.921) (RS-EGFR), 0.816 (0.645-0.916) (RS-19), 0.814 (0.595-0.938) (RS-21) Internal Validation: 0.780 (0.716-0.917) (RS-EGFR), 0.789 (0.636-0.942) (RS-19), 0.770 (0.478-0.919) (RS-21) External Validation: 0.807 (0.714-0.914) (RS-EGFR), 0.742 (0.609-0.931) (RS-19), 0.792 (0.530-0.946) (RS-21)	Radiomics-based MRI features were used to predict EGFR mutation subtypes in spinal metastases from lung adenocarcinoma, demonstrating good performance across validation cohorts.
8	Development and externally validate MRI-based nomogram to assess EGFR and T790M mutations in patients with metastatic lung adenocarcinoma	2022	Fan, Y. et al. [25]	Lung Adenocarcinoma with Spinal Metastasis	192 (160 primary + 32 external)	EGFR mutation (including T790M resistance mutation)	Machine Learning (Radiomics-based nomogram model)	No	Yes (T1-weighted, T2-weighted fat-suppressed MRI)	EGFR mutation and T790M resistance mutation prediction	Training: 0.849 (EGFR), 0.842 (T790M) Internal Validation: 0.828 (EGFR), 0.823 (T790M) External Validation:	Training: 0.685 (EGFR), 0.750 (T790M) Internal Validation: 0.964 (EGFR), 0.667 (T790M) External Validation: 0.611 (EGFR), 0.875 (T790M)	Training: 0.885 (EGFR), 0.867 (T790M) Internal Validation: 0.692 (EGFR), 0.938 (T790M) External Validation: 0.929 (EGFR), 0.800 (T790M)	Training (k-fold cross validation): 0.849 (0.776-0.922) (EGFR), 0.842 (0.717-0.927) (T790M) Internal Validation: 0.828 (0.708-0.949) (EGFR),	Radiomics-based MRI features from spinal metastases were used to predict both EGFR and T790M resistance mutations in lung adenocarcinoma. Nomogram

											0.778 (EGFR), 0.800 (T790M)			0.823 (0.633-0.940) (T790M)<br>External Validation: 0.778 (0.610-0.946) (EGFR), 0.800 (0.548-0.948) (T790M)	models incorporating radiomics and clinical factors (smoking status) demonstrated strong predictive performance.
9	MRI-Based Radiomics Nomogram as a Potential Biomarker to Predict the EGFR Mutations in Exon 19 and 21 Based on Thoracic Spinal Metastases in Lung Adenocarcinoma	2022	Cao, R. et al. [26]	Lung Adenocarcinoma (Spinal Bone Metastases)	76	EGFR (Exon 19, Exon 21)	Machine Learning (Radiomics)	No	Yes	Differentiation of EGFR Mutation Subtypes (Exon 19 vs. Exon 21)	Combined radiomics signature: 0.74 / 0.81, Clinical model: 0.56 / 0.69, Nomogram: 0.82 / 0.81	Combined radiomics signature: 0.76 / 0.69, Clinical model: 0.72 / 0.77, Nomogram: 0.92 / 0.85	Combined radiomics signature: 0.84 / 0.92, Clinical model: 0.92 / 0.69, Nomogram: 0.80 / 0.85	TRAINING (k-fold cross validation): Combined radiomics model: 0.867 (0.741-0.947); Clinical model :0.586 (0.438-0.723); Nomogram: 0.901 (0.783-0.967)<br><br>Internal VALIDATION: Combined radiomics signature: 0.864 (0.673-0.966); Clinical model :0.716 (0.507-0.874); Nomogram: 0.882 (0.695-0.974)	Developed a radiomics nomogram integrating MRI features and clinical factors for predicting EGFR mutation subtypes in spinal metastases.
10	Subregional radiomics analysis for the detection of the EGFR mutation on thoracic spinal metastases from lung cancer	2021	Fan, Y. et al. [27]	Lung Adenocarcinoma (Thoracic Spinal Metastases)	94	EGFR Mutation	Machine Learning (LASSO Regression)	No	Yes	EGFR Mutation Detection	Training group.<br>T1W-S1 0.656<br>T1W-S2 0.661<br>T1W-S3 0.694<br>T1W-W<br>0.694<br><br>Test group.<br>T1W-S1 0.516<br>T1W-S2 0.594<br>T1W-S3 0.656<br>T1W-W 0.625<br><br>Training group.	Training group.<br>T1W-S1 0.611<br>T1W-S2 0.757<br>T1W-S3 0.811<br>T1W-W 0.784<br><br>Test group.<br>T1W-S1 0.722<br>T1W-S2 0.526<br>T1W-S3 0.526<br>T1W-W 0.737<br><br>Training group.<br>T1W-S1 0.919<br>T1W-S2 0.605<br>T1W-S3 0.784	Training group.<br>T1W-S1 0.840<br>T1W-S2 0.680<br>T1W-S3 0.720<br>T1W-W<br>0.680<br><br>Test group.<br>T1W-S1 0.823<br>T1W-S2 0.769<br>T1W-S3 1.000<br>T1W-W 0.615<br><br>Training group.<br>T1W-S1 0.600<br>T1W-S2 0.760	Training group.<br>T1W-S1 0.720 (0.592–0.849)<br>T1W-S2 0.764 (0.640–0.889)<br>T1W-S3 0.786 (0.667–0.905)<br>T1W-W 0.758 (0.629–0.887)<br><br>Test group.<br>T1W-S1 0.611 (0.405–0.818)<br>T1W-S2 0.623 (0.422–0.826)	MRI-based radiomics analysis of spinal metastases subregions demonstrated potential for EGFR mutation detection. The inner subregion S3 showed the best detection performance.

											T1W-S1 0.693<br>T1W-S2 0.651<br>T1W-S3 0.726<br>T1W-W<br>0.710<br><br>Test group.<br>T1W-S1 0.563<br>T1W-S2 0.531<br>T1W-S3 0.688<br>T1W-W 0.656	T1W-W<br>0.757<br><br>Test group.<br>T1W-S1 0.737<br>T1W-S2 0.626<br>T1W-S3 0.895<br>T1W-W 0.737	T1W-S3 0.800<br>T1W-W<br>0.760<br><br>Test group.<br>T1W-S1 0.769<br>T1W-S2 0.846<br>T1W-S3 0.615<br>T1W-W 0.769	T1W-S3 0.713 (0.527–0.898)<br>T1W-W 0.623 (0.410–0.837)<br><br>Training group.<br>T2FS-S1 0.791 (0.669–0.912)<br>T2FS-S2 0.708 (0.578–0.839)<br>T2FS-S3 0.838 (0.741–0.935)<br>T2FS-W 0.797 (0.679–0.914)<br><br>Test group.<br>T2FS-S1 0.660 (0.456–0.864)<br>T2FS-S2 0.607 (0.407–0.808)<br>T2FS-S3 0.761 (0.583–0.940)<br>T2FS-W 0.704 (0.516–0.893)<br><br>Multi-Regional Radiomics Signature Model:<br>Training (k-fold cross validation):<br>AUC 0.879 (0.766-0.947)<br>Test (internal validation):<br>AUC 0.777 (0.612-0.967)	
11	MRI-based radiomics analysis for predicting the EGFR mutation based on thoracic spinal metastases in lung adenocarcinoma patients	2021	Ren, M.et al. [28]	Lung Adenocarcinoma (Thoracic Spinal Metastases)	162 (110 Training + 52 Validation)	EGFR Mutation	Machine Learning (Various Classifiers) + Deep Learning Features	No	Yes (T1W, T2W, T2FS)	EGFR Mutation Detection	NA	TRAINING: Combined Radiomics Signature : 0.935<br>Nomogram: 0.839<br><br>VALIDATION: Combined	TRAINING: Combined Radiomics Signature : 0.688<br>Nomogram: 0.792<br><br>VALIDATION: Combined	TRAINING (k-fold cross validation): Combined Radiomics Signature : 0.886 (95% confidence interval [CI]: 0.826-0.947)	A multi-parametric MRI-based radiomics model incorporating a combined radiomics signature and smoking status achieved high

												Radiomics Signature :0.700 Nomogram: 0.667	Radiomics Signature :0.818 Nomogram: 0.909	Nomogram: 0.888 (95% CI: 0.849-0.958)<br><br>Internal VALIDATION: Combined Radiomics Signature : 0.803 (95% CI: 0.682-0.924) Nomogram: 0.821 (95% CI: 0.692-0.929)	predictive accuracy for EGFR mutation.

Supplementary Table S3 – PROBAST Step 1 – specifying the systematic review question

**Criteria**	**Specify your systematic review question**
Intended use of model:	To aid in diagnosis, prognosis, and treatment decision-making for patients with spinal metastases, using AI-driven models and/or genomic/molecular predictors.
**Participants** including selection criteria and setting:	Adults (≥18 years old) diagnosed with spinal metastases, regardless of primary cancer origin. Studies may be conducted in tertiary hospitals, cancer centers, or multi-institutional registries. Inclusion criteria: studies that developed, validated, or applied prediction models (including ML/AI or molecular biomarker-based) to spinal metastasis. Exclusion: case reports, editorials, or studies without model development or validation.
**Predictors** (used in prediction modelling), including types of predictors (e.g. history, clinical examination, biochemical markers, imaging tests), time of measurement, specific measurement issues (e.g., any requirements/ prohibitions for specialized equipment):	- Types: Genomic/molecular biomarkers (e.g., TP53, PTEN, VEGF), clinical data (e.g., age, performance status), radiologic features, and AI-derived inputs.<br>- Time of Measurement: At initial diagnosis of spinal metastasis or prior to treatment planning.<br>- Measurement Issues: May require access to high-throughput genomic sequencing, bioinformatics platforms, or machine learning infrastructure. Standardization may vary across studies, particularly in imaging and molecular techniques.
Outcome to be predicted	- Diagnostic: Presence or likelihood of spinal metastasis.<br>- Prognostic: Overall survival, progression-free survival, neurologic deterioration, or spinal instability risk.<br>- Predictive: Response to treatment modalities (surgery, radiotherapy, systemic therapy).<br>- Model Performance: Accuracy, AUC, calibration, decision curve analysis.

Supplementary Table S4 – PROBAST Step 2 – classifying the type of prediction model evaluation

**Classify the evaluation based on its aim**			
**Type of prediction studies**	**PROBAST boxes to complete**	**Indicate as appropriate**	**Definition for type of prediction model study**
Development only	Development		Prediction model development without external validation. These studies may include internal validation methods, such as bootstrapping and cross-validation techniques.
Development and validation	Development and validation		Prediction model development combined with external validation in other participants in the same article.
Validation only	Validation		External validation of existing (previously developed) model in other participants.

PROBAST Step 3 – Assessing risk of bias and applicability

Four key domains, each judged for risk of bias (low, high or unclear). Each domain has signalling questions rated as yes (Y), probably yes (PY), probably no (PN), no (N) or no information (NI). All signalling questions are phrased so that "yes" indicates absence of bias. Any signalling question rated as "no" or "probably no" flags the potential for bias; you will need to use your judgement to determine whether the domain should be rated as "high", "low" or "unclear" risk of bias.

Domain 1: Participants

1.1 Were appropriate data sources used, e.g. cohort, RCT or nested case-control study data?

1.2 Were all inclusions and exclusions of participants appropriate?

Domain 2: Predictors

2.1 Were predictors defined and assessed in a similar way for all participants?

2.2 Were predictor assessments made without knowledge of outcome data?

2.3 Are all predictors available at the time the model is intended to be used?

Domain 3: Outcome

3.1 Was the outcome determined appropriately

3.2 Was a prespecified or standard outcome definition used?

3.3 Were predictors excluded from the outcome definition?

3.4 Was the outcome defined and determined in a similar way for all participants?

3.5 Was the outcome determined without knowledge of predictor information?

3.6 Was the time interval between predictor assessment and outcome determination appropriate?

Domain 4: Analysis

4.1 Were there a reasonable number of participants with the outcome?

4.2 Were continuous and categorical predictors handled appropriately?

4.3 Were all enrolled participants included in the analysis?

4.4 Were participants with missing data handled appropriately?

4.5 Was selection of predictors based on univariable analysis avoided?

4.6 Were complexities in the data (e.g. censoring, competing risks, sampling of controls) accounted for appropriately?

4.7 Were relevant model performance measures evaluated appropriately?

4.8 Were model overfitting and optimism in model performance accounted for?

4.9 Do predictors and their assigned weights in the final model correspond to the results from multivariable analysis?

RoB – overall risk of bias (high (H)/unclear (U)/low (L))

Applicability – overall concerns regarding applicability (high (H)/unclear (U)/low (L))

Supplementary Figure S1 – Risk of bias

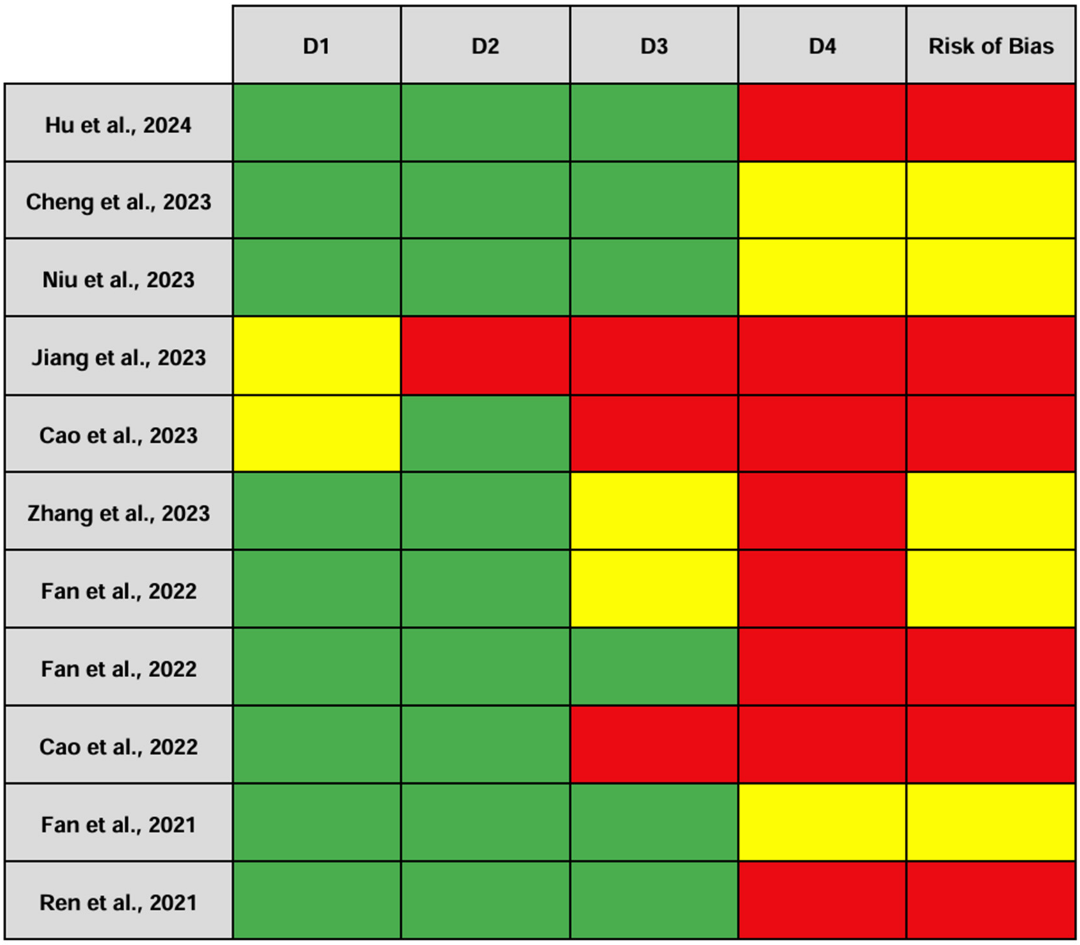

Domains:

D1: Bias introduced by selection of participants.
D2: Bias introduced by predictors or their assessment.
D3: Bias introduced by the outcome or its determination.
D4: Bias introduced by the analysis.

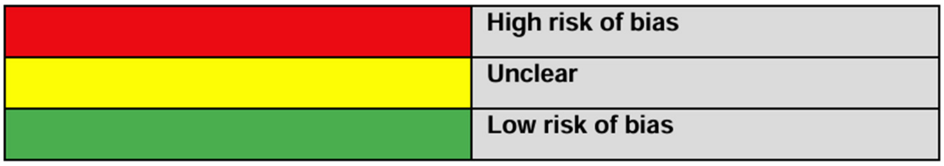

Supplementary Figure S2 – Applicability

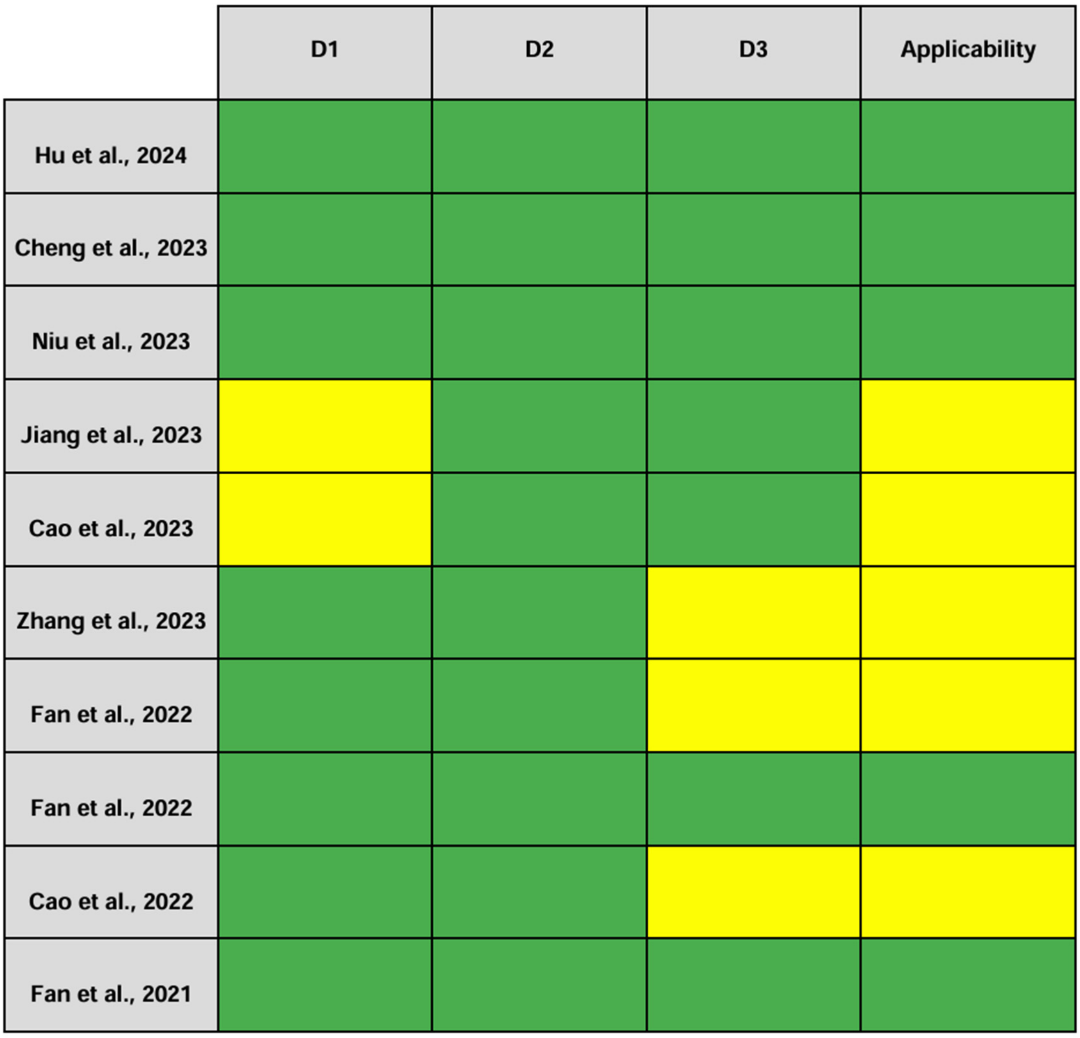

Domains:

D1: Applicability concern regarding the included participants and setting.
D2: Applicability concern regarding the definition, assessment or timing of prediction.
D3: Applicability concern regarding the outcome, its definition, timing or determination.

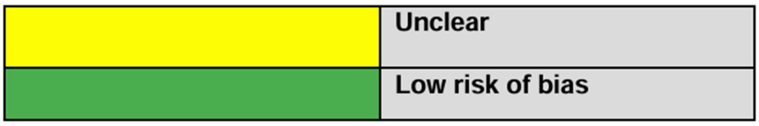

Supplement: Supplementary file 1 [file bioengineering-12-00791-s001.zip › bioengineering-3721688-supplementary.pdf]
